# Assessing the Quality of Operative Notes in the Orthopedic Surgery Department at Gezira Traumatology Center, Sudan, in Accordance With the Royal College of Surgeons Guidelines

**DOI:** 10.7759/cureus.89492

**Published:** 2025-08-06

**Authors:** Ammar Alemam Diab Alnour, Mirghani E Hussien, Ahmed Alhaj, Osman Faroug Mohammedosman Elhassan, Ahmed Eltahir, Mohamed Omer Emam, MohammedElhassan Abdalla

**Affiliations:** 1 Trauma and Orthopedics, Doncaster and Bassetlaw Teaching Hospitals NHS Foundation Trust, Doncaster, GBR; 2 Trauma and Orthopedics, Gezira Traumatology Center, Wad Madani, SDN; 3 Medicine and Surgery, University of Bahri, Khartoum, SDN; 4 Surgery, Our Lady of Lourdes Hospital Drogheda, Royal College of Surgeons in Ireland (RCSI) Hospital Group, Drogheda, IRL; 5 Surgery, University of Khartoum, Khartoum, SDN; 6 Orthopedic Surgery, Sudan Medical Specialization Board, Wad Madani, SDN

**Keywords:** clinical audit, documentation, orthopedic, quality improvement, surgical operative notes

## Abstract

Background: High-quality operative notes are crucial for patient safety, continuity of care, and medico-legal protection. Despite established Royal College of Surgeons (RCS) guidelines, audits globally reveal persistent deficiencies in surgical documentation. This study assessed the quality of operative notes in the Orthopedic Surgery Department at Gezira Traumatology Center, Wad Madani Teaching Hospital, Sudan, and evaluated the impact of targeted interventions.

Methods: A clinical audit was conducted in two cycles (in July 2023 and then three months post-intervention). In the first cycle, 23 randomly selected orthopedic operative notes were retrospectively audited against 14 RCS documentation parameters. Root cause analysis identified key deficiencies. A multi-faceted intervention was implemented, including staff education, a standardized RCS-aligned template, visual reminders, and a feedback mechanism. The second cycle re-audited 23 notes using the same criteria. Data were analyzed using descriptive statistics.

Results: Pre-intervention, compliance was high for procedure (95.7%), diagnosis (95.7%), and signature (100%), but critically low for theatre anesthetist's name (4.3%), anticipated blood loss. (13%), and antibiotic prophylaxis (8.7%). Post-intervention, significant improvements were noted in documenting the anesthetist's name (82.6%), anticipated blood loss (82.6%), complications (91.3%), and antibiotic prophylaxis (69.6%). However, unexpected declines were observed in documenting prosthesis identification (from 87% to 26.1%) and tissue details (from 87% to 65.2%). High-compliance areas generally remained stable.

Conclusion: Implementing a standardized operative note template based on RCS guidelines, combined with education and feedback, significantly improved the completeness of critical documentation elements in orthopedic operative notes. The decline in documenting prosthesis and tissue details highlights the need for focused reinforcement and ongoing monitoring to achieve consistent, comprehensive compliance. Standardized templates are highly effective tools for enhancing surgical documentation quality.

## Introduction

A well-structured operative note is fundamental to patient safety, effective postoperative care, and medico-legal protection in surgical practice [1]. Operative notes serve as a vital means of communicating surgical findings, procedure details, and postoperative instructions, thereby ensuring continuity of care and providing a reliable legal record [2]. Inadequate or incomplete documentation can lead to miscommunication, jeopardize patient management, and increase the risk of adverse outcomes, including morbidity and mortality [3]. Furthermore, accurate operative notes are essential for medico-legal defence and appropriate remuneration for surgical procedures [4]. To address the variability and deficiencies in operative note documentation, the Royal College of Surgeons (RCS) published comprehensive guidelines in 2014 as part of their "Good Surgical Practice" document [1]. These guidelines specify the essential components that should be included in every operative note, such as patient identification, date and time of surgery, names of the operating surgeon and assistants, name of the anesthetist, operative diagnosis and findings, details of the procedure, complications, details of any prosthesis or materials used, wound closure technique, anticipated blood loss, antibiotic and deep vein thrombosis (DVT) prophylaxis, detailed postoperative instructions, and the surgeon's signature [2,3]. The RCS guidelines are concise, targeted, and adaptable to any surgical specialty, making them a widely accepted standard for surgical documentation [3].

Despite the availability of these guidelines, audits conducted in various hospitals, including those in resource-limited settings, have identified persistent gaps in operative note completeness and accuracy [5,6]. For example, a study at the District Headquarter Hospital in Rawalpindi, Pakistan, demonstrated that adherence to RCS guidelines significantly improved the quality of operative notes, with notable increases in the documentation of operative diagnosis, names of surgical team members, and postoperative instructions following guideline implementation [5]. Similar findings were reported in audits from India, highlighting the importance of standardized templates and regular audits in improving documentation practices [7,8].

Recent research from Sudan, specifically at Dongola Teaching Hospital, has also shown that structured interventions based on RCS guidelines can enhance the quality of operative notes in general surgery departments [9]. A related audit at Port Sudan Teaching Hospital further corroborates these findings, demonstrating that targeted interventions and adherence to RCS standards can lead to significant improvements in the completeness and quality of surgical operative notes, even in challenging resource environments [10]. Moreover, a two-phase audit conducted at Elobeid Teaching Hospital provides additional evidence that systematic evaluation and continuous improvement strategies effectively raise operative note quality, supporting the generalizability of these findings across multiple Sudanese teaching hospitals [11]. Additional guidance and clinical standards can be found in the broader RCS clinical guidelines, further supporting the need for standardized documentation [4].

This evidence supports the broader application of RCS standards in diverse clinical settings, including orthopedic surgery. Thus, the present study aimed to assess the quality of operative notes in the Orthopedic Surgery Department at Gezira Traumatology Center, Wad Madani Teaching Hospital, Sudan, in July 2023, using the RCS guidelines as the reference standard [8].

## Materials and methods

Pre-intervention phase

In the pre-intervention phase, a retrospective review of operative notes was conducted in the Orthopedic Surgery Department at Gezira Traumatology Center. A total of 23 operative notes from July 2023 were randomly selected utilizing a random sampling technique and assessed for compliance with the RCS guidelines. Each note was evaluated for 14 key parameters, including patient identification, date and time, names of the surgical team, operative diagnosis, procedure details, complications, and postoperative instructions. The baseline results revealed variable compliance, with high adherence in some areas (e.g., operative procedure, diagnosis, and signature) and notable deficiencies in others, such as documentation of anticipated blood loss, antibiotic prophylaxis, and the name of the theatre anesthetist.

Root cause examination

A root cause analysis was conducted to explore the underlying reasons for incomplete or inconsistent surgical documentation. The assessment identified several key factors: a general lack of awareness among some surgical staff regarding the comprehensive documentation requirements outlined by the RCS; the absence of standardized templates, which resulted in variability in operative note formats and the omission of essential parameters; and significant time constraints, where high workloads and procedural pressures often led to rushed or partially completed entries. Additionally, limited feedback mechanisms meant that errors and omissions were rarely identified or corrected in a systematic manner. Inconsistent training also contributed, as new staff and trainees did not consistently receive structured orientation on documentation standards, further perpetuating gaps in compliance.

Intervention phase

Based on insights gained during the pre-intervention phase and the root cause analysis, a comprehensive, multi-pronged intervention was implemented to enhance compliance with operative note documentation standards. Educational sessions were conducted for surgeons, residents, and operating room staff, emphasizing the importance of thorough documentation and reviewing the RCS guidelines. To promote consistency, a standardized operative note template was developed in alignment with RCS standards and disseminated across relevant departments. Additionally, visual reminders in the form of posters outlining key documentation parameters were strategically placed in operating theatres and staff areas to reinforce daily practice. To ensure ongoing quality improvement, a structured feedback mechanism was introduced, enabling senior staff to regularly review and provide constructive input on operative notes.

Post-intervention phase

Three months after the intervention, the second cycle of the audit was conducted. Another 23 operative notes were randomly selected and assessed using the same criteria. Post-intervention results showed significant improvements in several parameters, particularly in the documentation of the name of the theatre anesthetist (from 4.3% to 82.6%), anticipated blood loss (from 13% to 82.6%), problems/complications (from 52.2% to 91.3%), and antibiotic prophylaxis (from 8.7% to 69.6%). However, a decline was noted in the documentation of identification of prosthesis used (from 87% to 26.1%) and details of tissue removed/added/altered (from 87% to 65.2%).

Data analysis

The analysis of data was facilitated by Microsoft Excel 2016 (Microsoft Corp., Redmond, USA), utilizing descriptive statistics such as frequencies and percentages to summarize compliance rates. A comparative analysis was also undertaken to discern patterns and prevalent shortcomings in documentation practices, thereby highlighting opportunities for enhancement.

## Results

A total of 23 operative records were reviewed in both the first and second cycles of this clinical audit. The quality of documentation improved significantly across several key domains following the introduction of targeted interventions.

In the first cycle, compliance with key documentation parameters varied, with notable deficiencies in certain areas. The name of the theatre anesthetist was recorded in just one (4.3%) operative note, anticipated blood loss in three (13%), and antibiotic prophylaxis in two (8.7%). Documentation of problems or complications was included in 12 (52.2%) notes, while details of tissue removed, added, or altered were present in 20 (87%), and identification of the prosthesis used was included in 20 (87%). The rationale for extra procedures was not specifically listed among your parameters, but several other parameters, such as incision, operative diagnosis, and closure technique, showed relatively high baseline compliance.

Following the implementation of documentation improvement strategies, particularly the introduction of a standardized template based on the RCS guidelines, the second cycle demonstrated substantial progress. Staff training sessions were also conducted, but the primary purpose of improvement was the use of the standardized template, which enhanced the completeness and accuracy of surgical operative notes. Documentation rates for the name of the theatre anesthetist improved dramatically from one (4.3%) to 19 (82.6%). Anticipated blood loss recording increased from three (13%) to 19 (82.6%), and antibiotic prophylaxis rose from two (8.7%) to 16 (69.6%). Documentation of problems or complications also improved, from 12 (52.2%) to 21 (91.3%).

Areas that already demonstrated high baseline compliance, such as operative procedure (95.7% in both cycles), operative diagnosis (95.7% to 91.3%), and detailed postoperative care instructions (100% to 95.7%), remained strong, confirming that these elements are well-established in routine practice. However, some parameters showed unexpected declines: identification of the prosthesis used decreased from 20 (87%) to six (26.1%), and details of tissue removed, added, or altered fell from 20 (87%) to 15 (65.2%). Incision documentation also decreased from 22 (95.7%) to 19 (82.6%), and closure technique documentation declined from 19 (82.6%) to 17 (73.9%).

The overall compliance trends indicated a marked enhancement in the completeness of operative records for most key parameters following the interventions (Table 1). 

**Table 1:** The documentation compliance rates from both the first and second audit cycles are presented, highlighting the extent of improvement between them.

Parameters	First cycle, n (%)	Second cycle, n (%)
Date and time	21 (91.3%)	20 (87%)
Name of operating surgeon and assistant	20 (87%)	22 (95.7%)
Name of theatre anesthetist	1 (4.3%)	19 (82.6%)
Operative procedure carried out	22 (95.7%)	22 (95.7%)
Incision	22 (95.7%)	19 (82.6%)
Operative diagnosis	22 (95.7%)	21 (91.3%)
Problems/complications	12 (52.2%)	21 (91.3%)
Details of tissue removed/added/altered	20 (87%)	15 (65.2%)
Identification of prosthesis used	20 (87%)	6 (26.1%)
Details of closure technique	19 (82.6%)	17 (73.9%)
Anticipated blood loss	3 (13%)	19 (82.6%)
Antibiotic prophylaxis	2 (8.7%)	16 (69.6%)
Detailed postoperative care instructions	23 (100%)	22 (95.7%)
Signature	23 (100%)	22 (95.7%)

The comparative analysis of operative documentation compliance rates, presented in Table 2, demonstrated a measurable improvement following the introduction of standardized interventions between the first and second audit cycles. Notably, mean compliance rose from 82.0% to 83.4%, with several parameters, such as theatre anesthetist identification, anticipated blood loss estimation, and antibiotic prophylaxis, showing substantial gains. Despite minor regressions in documentation of prosthesis details and incision technique, the overall trend supported the effectiveness of the implemented documentation template and targeted staff training. 

**Table 2:** Comparative compliance rates of operative documentation parameters between first and second audit cycles. The improvement of +1.4% in mean compliance reflects overall progress after documentation interventions, even accounting for fluctuations in specific parameters.

Cycle	Mean compliance (%)
First cycle	82.00%
Second cycle	83.40%

## Discussion

This clinical audit shows that targeted interventions, especially using a standardised operative note template that follows the RCS guidelines, made documentation much more complete for the most important parameters in orthopedic surgery at Wad Madani Teaching Hospital. The huge increases in recording the theatre anesthetist's name (from 4.3% to 82.6%), expected blood loss (from 13% to 82.6%), and antibiotic prophylaxis (from 8.7% to 69.6%) show how effective structured tools can be at breaking down barriers like inconsistent knowledge of documentation standards and the lack of systematic feedback mechanisms. This is similar to how template-based interventions increased compliance by 30-85% in Sudan and South Asia [5,7,9]. The fact that almost all of the people who experienced complications after the intervention (91.3%) documented them shows how combining education with practical tools makes it easier to follow through, even when time is limited in the clinic.

However, an unexpected result emerged: the number of people who could identify a prosthesis dropped from 87% to 26.1%, and the number of people who could describe how the tissue changed fell from 87% to 65.2%. This paradox is likely the result of cognitive overload as staff learned new areas, template design flaws that pushed less important sections to the background (such as prosthetics documentation that was buried in dense text), or selective attention, where new parameters that were given more weight overshadowed established ones [12]. The fact that both the incision (95.7% to 82.6%) and closure technique (82.6% to 73.9%) declined at the same time suggests that rigid templates can mess up established documentation habits if they aren't designed to be easy to use. This is similar to the problems faced at Port Sudan Teaching Hospital, where the "implants used" fields were still filled out inconsistently even after standardisation [7,10,11]. The partial improvement in antibiotic prophylaxis (69.6%) is significant, but it also shows that there are still gaps in the system, which could be due to unclear accountability or insufficient training on protocols [4,12].

These results show that even though workshops and visual reminders helped address some of the root causes, such as template variability and time pressures [5,9,12], there are still gaps that require better strategies to fill them. Redesigning templates should prioritize high-risk parts (like prostheses and tissue details) at the top of the list, with checkboxes and large spaces to ensure they don't get missed. Adding dropdown menus for antibiotic regimens could make it easier to enter complicated information [4,10]. Training must require RCS modules during onboarding and annual evaluations, especially for junior staff who tend to miss more [11]. Real-time feedback systems, such as senior reviews within 24 hours, which were tested in Lahore with more than 90% compliance, could help fix mistakes right away [5]. Digital solutions, such as mandatory fields in electronic health records (EHRs) with automatic reminders, appear promising for places that can manage the technology [7].

Recent findings from Doka Hospital further support this narrative. In their closed-loop audit, Hassan Ibrahim et al. [13] implemented a pro forma based on RCS guidelines and observed an impressive compliance improvement from 50.5% to 82.5% across 100 cases per cycle. The study underscores the effectiveness of structured note-taking formats combined with targeted training. However, despite dramatic gains in areas such as closure technique (from 48% to 100%) and tissue documentation (from 25% to 85%), prosthesis identification remained stagnant at 0%, echoing gaps observed elsewhere and reinforcing the need for improvements in interface design.

Study limitations 

This study has several limitations that should be acknowledged. The small sample size (n=23 per cycle) and single-center design may restrict the generalizability of the findings, though they reflect pragmatic constraints common in rapid-cycle audits. The short three-month post-intervention period also limits the assessment of long-term sustainability. Larger multicenter audits with extended follow-up (6-12 months), such as the Elobeid Teaching Hospital’s two-phase study [11], would be valuable to evaluate compliance decay over time. Additionally, the absence of inferential statistical testing and detailed methodological reporting (e.g., randomization process, intervention specifics) may affect reproducibility. Potential biases, including observer bias or Hawthorne effects, were not explicitly examined. Finally, the lack of qualitative feedback from surgeons, particularly regarding practical challenges like template usability, represents a missed opportunity to identify barriers to implementation. Future work should incorporate structured interviews or surveys to capture these insights and further refine intervention strategies.

## Conclusions

In conclusion, RCS-aligned templates and education are powerful tools for improving surgical documentation in places with few resources. However, their design must change over time to avoid losing important parts. To ensure continued success, there is a need to test user-centered template modifications, conduct compliance audits every six months with feedback loops, and incorporate documentation standards into surgical safety checklists. Standardization without adaptability carries the risk of replacing old errors with new ones. Ultimately, frontline-driven refinement is the most important thing for operative notes to fulfill their role in ensuring patient safety, continuity of care, and maintaining both medical and legal integrity.
